# Annual Meeting of the Erie County Medical Society

**Published:** 1872-01

**Authors:** 


### Editorial Department.

### Annual Meeting of the Erie County Medical Society.---Reception of a Portrait of Dr. Levi J. Ham.

The annual meeting of the Erie County Medical Society was held in Buffalo Tuesday, Jan. 9, and was quite largely attended. The election of officers and propositions to amend the by-laws of the Society, occupied most of the time of the session. Some amendments were passed (supposed to be passed) but as there had been no previous written notice of intention to amend, the action was irregular, and must come up for future approval. The regular order of business was suspended by motion of Dr. Stork, when Dr. Miner read the following communication:

South Bend, Ind., Dec. 22, 1871.

My Dear Doctor:—I ship my portrait to the Erie County Medical Soceity, to your care, per Express to-day. Please present it to the Society in my name, as a token of the kindly regards I still entertain for it, and for its individual members. The study and practice of my profession has been a life business with me; I still love it. Respects to all the members of the Society.

Yours, very truly,

J. F. Miner, M. D. LEVI J. HAM.

Accompanying this he has furnished the following particulars of his life:

Born Nov. 16, 1805. Graduated in Dartmouth College August 28, 1828. Received Medical Degree Bowdoin College March 19, 1831. Was a private student of Prof. R. D. Mussey two years. Practiced in York county, Maine, from 1831 to 1845. Practiced in Williamsville, Erie county, N. Y., from 1846 to 1859. Practiced in South Bend, Ind., from 1859 to 1871. Was appointed Surgeon of the 48th Ind. vol. Dec. 4, 1861. Resigned April 9, 1865. Was

Chairman of the Operating Board of Surgeons in the 7th Division of the 17th Army Corps in all the great battles in front of Vicksburgh 1863-4, and at Lookout Mountain and Mission Ridge in 1864. Was at one time Medical Director of the 17th Army Corps under the gallant McPherson.

He says: "I have always had a taste for operative surgery; always been eminently conservative in all my operations. I now have a good practice, a good home, good friends, and a competence of this world's goods."

To this brief and expressive autobiography of Dr. Ham, it is proper that I add, that he has always held high and honorable position in his profession, and that he is rightfully regarded by both the profession and public as a high minded, honorable physician, whose ample opportunities of early education, and whose extensive observation and experience have fitted for professional eminence. His kind mention of his former membership with us, and the generous donation of his portrait adds to the number of his numerous acts which are worthy of imitation Especially is this generous, acceptable, and appropriate donation of his portrait worthy of imitation, by his former associates, many of whom, will soon no more return to grace these annual meetings with their actual presence, and who may thus help to keep alive memories which all desire to cherish.

Dr. Edward Stork made a very pleasant reference to the character and surgical skill of Dr. Ham while practicing in Erie county, and *moved* "that the portrait be accepted and the thanks of the Society returned, with hearty good wishes for his health, happiness and long continued usefulness."

The reception of a large, full-sized portrait, of a much respected former member, was occasion of much gratification to the Society.

